# Perinatal mood and anxiety disorders, serious mental illness, and delivery-related health outcomes, United States, 2006–2015

**DOI:** 10.1186/s12905-020-00996-6

**Published:** 2020-07-23

**Authors:** Kimberly McKee, Lindsay K. Admon, Tyler N. A. Winkelman, Maria Muzik, Stephanie Hall, Vanessa K. Dalton, Kara Zivin

**Affiliations:** 1 Department of Family Medicine, University of Michigan, 1018 Fuller Street, Ann Arbor, MI 48104-1213 USA; 2 Department of Obstetrics & Gynecology, University of Michigan, Ann Arbor, MI USA; 3 University of Michigan Institute for Healthcare Policy and Innovation, Ann Arbor, MI USA; 4 Program on Women’s Healthcare Effectiveness Research, University of Michigan, Ann Arbor, MI USA; 5 Division of General Internal Medicine, Hennepin Healthcare, Minneapolis, MN USA; 6 Hennepin Healthcare Research Institute, Minneapolis, MN USA; 7 Department of Psychiatry, University of Michigan, Ann Arbor, MI USA; 8 Department of Learning Health Sciences, University of Michigan, Ann Arbor, MI USA; 9 VA Ann Arbor Healthcare System, Ann Arbor, MI USA; 10 Department of Health Policy & Management, School of Public Health, University of Michigan, Ann Arbor, MI USA

**Keywords:** Perinatal mood and anxiety disorders, Serious mental illness, Depression/anxiety, Pregnancy, Severe maternal morbidity and mortality

## Abstract

**Background:**

National estimates of perinatal mood and anxiety disorders (PMAD) and serious mental illness (SMI) among delivering women over time, as well as associated outcomes and costs, are lacking. The prevalence of perinatal mood and anxiety disorders and serious mental illness from 2006 to 2015 were estimated as well as associated risk of adverse obstetric outcomes, including severe maternal morbidity and mortality (SMMM), and delivery costs.

**Methods:**

The study was a serial, cross-sectional analysis of National Inpatient Sample data. The prevalence of PMAD and SMI was estimated among delivering women as well as obstetric outcomes, healthcare utilization, and delivery costs using adjusted weighted logistic with predictive margins and generalized linear regression models, respectively.

**Results:**

The study included an estimated 39,025,974 delivery hospitalizations from 2006 to 2015 in the U.S. PMAD increased from 18.4 (95% CI 16.4–20.0) to 40.4 (95% CI 39.3–41.6) per 1000 deliveries. SMI also increased among delivering women over time, from 4.2 (95% CI 3.9–4.6) to 8.1 (95% CI 7.9–8.4) per 1000 deliveries. Medicaid covered 72% (95% CI 71.2–72.9) of deliveries complicated by SMI compared to 44% (95% CI 43.1–45.0) and 43.5% (95% CI 42.5–44.5) among PMAD and all other deliveries, respectively. Women with PMAD and SMI experienced higher incidence of SMMM, and increased hospital transfers, lengths of stay, and delivery-related costs compared to other deliveries (*P* < .001 for all).

**Conclusion:**

Over the past decade, the prevalence of both PMAD and SMI among delivering women increased substantially across the United States, and affected women had more adverse obstetric outcomes and delivery-related costs compared to other deliveries.

## Background

Perinatal mood and anxiety disorders (PMAD), which encompass depression and anxiety, are among the most common conditions identified during pregnancy and the postpartum period. Serious mental illness (SMI) entails mental, behavioral, or emotional disorders that substantially impact functional impairment, and include bipolar and psychotic disorders [1, 2]. Untreated, mental health conditions in the perinatal period may lead to adverse outcomes for mothers and their children [3–5], including, preterm birth and maternal suicide, which is a leading cause of maternal morbidity and mortality [6]. Beyond the immediate perinatal period, the long-term effects on mothers and their families include reductions in infant cognitive and social-emotional development, behavior, and family functioning [7–9]. Although awareness around screening for PMAD has increased, ensuring there is appropriate follow-up and adherence to adequate treatment to remission for affected women is necessary to improve clinical outcomes [10]. Despite the significant impact of PMAD and SMI on maternal and child health, national estimates of the prevalence of these conditions and their impact on healthcare utilization and costs are understudied.

Given the recently documented temporal increase in chronic medical conditions among delivering women, and their contribution to rising maternal morbidity and mortality [11], we hypothesized that PMAD and SMI have increased nationally over time as well, and that they are associated with adverse birth outcomes and severe maternal morbidity and mortality (SMMM), greater health care utilization, and higher delivery costs. Therefore, in this paper we assessed national trends in the prevalence of PMAD and SMI among delivering women and associated obstetric outcomes including SMMM, healthcare utilization, and delivery-related costs. These data are critical to quantify the burden of mental health conditions among delivering women, identify women that would most benefit from evidence-based treatments, and prioritize allocation of limited resources in efforts to address rising maternal mortality and morbidity.

## Methods

### Study sample

The study conducted a serial, cross-sectional analysis using 2006–2015 data from the National Inpatient Sample (NIS) [12], the largest nationally representative sample of hospital deliveries in the United States. The NIS is a stratified sample of 20% of all discharges from community-based hospitals in the United States administered by the Agency for Healthcare Research and Quality’s Healthcare Cost and Utilization Project (HCUP). All analyses conform to the methodological standards for research using the NIS [13]. Sample weights allow for nationally representative estimates [14]. The study identified delivery hospitalizations using delivery codes in a hierarchical manner: (1) outcome of delivery (ICD-9-CM disease code = V27), (2) normal delivery (ICD-9-CM disease code = 650, (3) diagnosis-related group (DRG) delivery codes, and 4) ICD-9-CM procedure codes for selected delivery- related procedures based on previously published methods using NIS HCUP data [14].

### Independent variables

The study included PMAD and SMI identified from the delivery record at discharge using International Classification of Diseases, Ninth Revision, Clinical Modification (ICD-9-CM) codes (Table 1). Covariates included maternal age, payer (i.e., Medicaid, private insurance, or uninsured), ZIP code income quartile, rural compared with urban residence, and hospital census region. Location of residence included an indicator of rural or urban using the National Center for Health Statistics Classification and Urban Influence codes [15].

**Table 1:** International classification of diseases, ninth revision, clinical modification codes for perinatal mood and anxiety disorders and serious mental illness conditions

PMAD	Depression	Major depressive affective disorder 296.20, 296.21, 296.22, 296.23, 296.24, 296.25, 296.26, 296.30, 296.31, 296.32, 296.33, 296.34, 296.35, 296.36, Dysthymic disorder 300.4 Depressive disorder, not elsewhere classified 311
	Anxiety	Anxiety disorder 293.84, 300.00, 300.01, 300.02, 300.09, 300.10 Phobia 300.20, 300.21, 300.22, 300.23, 300.29 Obsessive-compulsive disorder 300.3 Neurasthenia and other somatoform disorders 300.5, 300.89, 300.9 Acute stress reaction 308.0, 308.1, 308.2, 308.3, 308.4, 308.9 Posttraumatic Stress Disorder 309.81 Overanxious disorder 313.0, 313.1, 313.21, 313.22, 313.30, 313.82, 313.83
SMI	Bipolar disorder	Bipolar I disorder 296.00, 296.01, 296.02, 296.03, 296.04, 296.05, 296.06, 296.40, 296.41, 296.42, 296.43, 296.44, 296.45, 296.46, 296.50, 296.51, 296.52, 296.53, 296.54, 296.55, 296.56, 296.60, 296.61, 296.62, 296.63, 296.64, 296.65, 296.66, 296.7 Manic disorder 296.10, 296.11, 296.12, 296.13, 296.14, 296.15, 296.16 Other and unspecified bipolar disorders 296.80, 296.81, 296.82, 296.89 Other and unspecified episodic mood disorder 296.90, 296.99
	Psychotic Disorders	Psychotic disorder 293.81, 293.82 Schizophrenia 295.00, 295.01, 295.02, 295.03, 295.04, 295.05, 295.10, 295.11, 295.12, 295.13, 295.14, 295.15, 295.10, 295.11, 295.12, 295.13, 295.14, 295.15, 295.20, 295.21, 295.22, 295.23, 295.24, 295.25, 295.30, 295.31, 295.32, 295.33, 295.34, 295.35, 295.40, 295.41, 295.42295.43, 295.44, 295.45, 295.50, 295.51, 295.52, 295.53, 295.54, 295.55295.60, 295.61, 295.62, 295.63, 295.64, 295.65, 295.70, 295.71, 295.72295.73, 295.74, 295.75, 295.80, 295.81, 295.82, 295.83, 295.84, 295.85, 295.90, 295.91, 295.92, 295.93, 295.94, 295.95 Paranoid state 297.0297.1297.2297.3297.8297.9 Depressive psychosis 298.0, 298.1, 298.2, 298.3, 298.4, 298.8, 298.9

The study included maternal race and ethnicity for the years in which these data appeared reliably available (2012–2015) [16]. Race and ethnicity categories included non-Hispanic white, non-Hispanic black, Hispanic, Asian/Pacific Islander, and American Indian/Alaska Native. The study also included identification of at least one comorbid substance use disorder [17, 18].

### Delivery-related outcomes

The study examined three types of delivery-related outcomes: 1) obstetric outcomes (cesarean delivery, preterm delivery, and SMMM), 2) healthcare utilization (need for hospital transfer, mean length of stay), and 3) hospital costs. Preterm delivery (delivery at less than 37 weeks’ gestation) identified from ICD-9-CM codes 644.21, and cesarean delivery identified from ICD-9-CM procedure codes 74.0, 74.1, 74.2, 74.4, 74.9x. SMMM identified from ICD-9-CM indicators outlined by the Centers for Disease Control and Prevention [19]. Mortality data came from the hospital discharge disposition in the NIS [20]. Transfers indicate that a patient was either transferred in for a delivery hospitalizations or out after a delivery hospitalization [21]. Delivery-related hospital costs were calculated using HCUP’s cost-to-charge ratio files [22], inflation-adjusted to 2015 U.S. dollars.

### Statistical analysis

The analysis calculated weighted frequencies for maternal socio-demographic characteristics across three categories: PMAD, SMI, and all other hospital deliveries. The approach used weighted logistic regression models with predictive margins to calculate the prevalence of PMAD and SMI per 1000 delivery hospitalizations for each two-year period. Subgroup analyses included maternal race/ethnicity using pooled data from 2012 to 2015.

Adjusted multivariable logistic regression models estimated delivery-related outcomes with and without PMAD/SMI. Generalized linear models with a log-link function and gamma distributions estimated mean length of stay and delivery-related costs. All models included covariate adjustments for maternal age, payer, ZIP code income quartile, rural residence, and hospital region. All estimates used weighted and post-regression predictive margins, tabulated per 100 delivery hospitalizations.

The 2015 data only used ICD-9-CM diagnoses codes for the first three quarters; thus, the 2015 survey weights adjusted to use annualized estimates from the first three quarters of data. All analyses used STATA 14.2. The University of Michigan Institutional Review Board considered this study exempt from review.

## Results

In total, the study identified 7,906,820 delivery hospitalizations, representing an estimated 39,025,974 deliveries that occurred between 2006 and 2015. Within this sample, 219,294 deliveries included PMAD (weighted *N* = 1,107,001), and 50,178 deliveries included SMI (weighted *N* = 251,381). Medicaid covered 72% (95% CI 71.2–72.9) of all deliveries complicated by SMI compared to 44% (95% CI 43.1–45.0) of deliveries with PMAD and 43.5% (95% CI 42.5–44.5) of all other deliveries (Table 2). Higher proportions of women with SMI lived in the lowest income quartile compared to women with PMAD and all other deliveries. Higher proportions of women with PMAD and SMI had > = 1 substance abuse disorder [19.1% (95% CI 18.6–19.6) and 37.3% (95% CI 36.5–38.1), respectively] compared to other deliveries [5.4% (95% CI 5.2–5.6)].

**Table 2:** Characteristics of delivering women with PMAD and SMI, national inpatient sample, 2006–2015 (Unweighted *N* = 7,906,820)^a^

	All Other Hospital Deliveries (unweighted N = 7,637,348)	PMAD (unweighted ***N*** = 219,294)	SMI (unweighted) ***N*** = 50,178)
	Weighted % (95% CI)	Weighted % (95% CI)	Weighted % (95% CI)
Age (mean, years)^b^	27.8 (27.7–27.9)	**29.7 (28.6–28.8)**	**26.5 (26.5–26.6)**
Insurance Payer			
Medicaid	43.5 (42.5–44.5)	**44.0 (43.1–45.0)**	**72.0 (71.2–72.9)**
Private	50.4 (49.3–51.5)	**51.2 (50.2–52.2)**	**23.5 (22.7–24.4)**
Uninsured	6.1 (5.7–6.5)	**4.8 (4.5–5.0)**	**4.4 (4.1–4.8)**
Income			
Bottom quartile^c^	27.6 (26.6–28.6)	**23.9 (23.0–24.8)**	**35.9 (34.8–37.1)**
Residence			
Rural	14.4 (13.8–15.1)	**15.8 (15.0–16.6)**	**16.0 (15.2–16.8)**
Hospital Region			
Northeast	15.9 (14.9–16.9)	**19.5 (18.0–21.0)**	**19.4 (18.0–20.8)**
Midwest	21.2 (20.0–22.5)	**27.0 (25.5–28.5)**	**25.4 (23.7–27.2)**
South	38.2 (36.5–39.9)	**31.2 (29.5–32.9)**	**36.6 (34.7–38.5)**
West	24.7 (23.3–26.2)	**22.4 (20.9–23.9)**	**18.7 (17.4–20.1)**
> 1 Co-morbid substance use disorder	5.4 (5.2–5.6)	**19.1 (18.6–19.6)**	**37.3 (36.5–38.1)**

^a^All proportions are represented as weighted % (95% Confidence Interval) unless otherwise noted. Boldface indicates statistical significance (*P* < 0.001)

^b^Weighted mean (95% Confidence Interval)

^c^Represents patients living in a ZIP code with a median household income in the bottom national income quartile

**Fig. 1:**
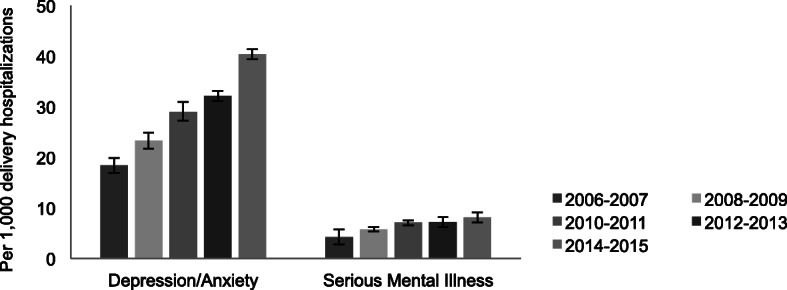
Trends in PMAD and SMI at Delivery in the United States, 2006–2015 (*N* = 7,906,820)^a^. The prevalence of both perinatal mood and anxiety disorders and serious mental illness among delivering women increased across the United States from 2006 to 2015

The prevalence of PMAD increased from [18.4 (95% CI 16.4–20.0)] to 40.4 (95% CI 39.3–41.6) per 1000 delivery hospitalizations] between 2006 and 07 and 2014–15. The prevalence of SMI also increased over time from [4.2 (95% CI 3.9–4.6)] to [8.1 (95% CI 7.9–8.4) per 1000 delivery hospitalizations] (Fig. 1).

The incidence of PMAD and SMI differed by race/ethnicity from 2012 to 2015. Non-Hispanic white women had higher prevalence of PMAD and SMI [75.3% (95% CI 3.9–4.6) and 67.6% (95% CI 66.6–68.5), respectively] compared to deliveries without either condition [55.4% (95% CI 54.7–56.0)]. Black women had a higher proportion of deliveries with SMI [21.1% (95% CI 20.2–22.0)] compared to those with PMAD [10.6% (95% CI 10.1–11.00] or neither [15.4% (95% CI 15.0–15.8)]. Hispanic and Asian/Pacific Islander women had a lower prevalence of PMAD [11.5% (95% CI 11.0–12.50) and 1.9% (95% CI 1.7–2.0), respectively] and SMI [9.4% (95% CI 8.9–10.0) and 1.1% (95% CI 0.9–1.3), respectively] at delivery compared to that among other hospital deliveries (22.3% (95% CI 21.7–22.9) and 6.1% (95% CI 5.9–6.4), respectively].

The incidence of preterm delivery was higher among women with PMAD [9.7 (95% CI 9.4–10.0)] and SMI [10.8 (95% CI 10.4–11.1)] compared to deliveries without either condition [6.7 (95% CI 6.7–7.0) per 100 deliveries]. Women with PMAD and SMI experienced greater incidence of SMMM [2.3 (95% CI 2.2–2.4)] and [2.1 (95% CI 1.9–2.2), respectively] compared to [1.5 (95% CI 1.5–1.5) per 100 deliveries] (Table 3) than those without PMAD and SMI. Deliveries with PMAD and SMI had more hospital transfers and longer lengths of stay, respectively, compared to those without either condition. Women with PMAD had higher mean delivery-related costs ($5200; 95% CI $5100-5200) and SMI ($5300; 95% CI $5200-5400) compared to other deliveries ($4400; 95% CI $4300-4500).

**Table 3:** Adjusted obstetric outcomes, healthcare utilization and expenditures among women with depression/anxiety and serious mental illness, 2006–2015 (*N* = 7,906,820)^a^

	All Other Hospital Deliveries (***n*** = 7,637,348)	PMAD (***n*** = 219,294)	SMI (***n*** = 50,178)
**Obstetric outcomes**			
Severe maternal morbidity/mortality	1.5 (1.5–1.5)	2.3 (2.2–2.4)	2.1 (1.9–2.2)
Preterm delivery^b^	6.7 (6.7–7.0)	9.7 (9.4–10.0)	10.8 (10.4–11.1)
Cesarean delivery	32.7 (32.4–33.0)	37.7 (37.3–38.1)	38.8 (38.2–39.2)
**Healthcare utilization**			
Hospital transfer	0.7 (0.7–0.8)	1.5 (1.4–1.6)	2.2 (2.0–2.4)
Mean Length of stay (days)^c^	2.6 (2.6–2.6)	3.1 (3.0–3.1)	3.1 (3.1–3.2)
**Healthcare expenditures**			
Mean cost per delivery hospitalization (USD, mean)^d^	4400 (4300-4500)	5200 (5100-5200)	5300 (5200-5400)

^a^All proportions are survey-weighted and represented as rate per 100 delivery hospitalizations (95% Confidence Interval) unless otherwise noted. Adjusted for maternal age, payer, ZIP code income quartile, rural residence, hospital region. ***P*** **< 0.001** for all comparisons

^b^Delivery at less than 37 weeks gestational age

^c^Means are reported with 95% confidence intervals

^d^Costs are inflation-adjusted to 2015 U.S. dollars (USD)

## Discussion

The national estimates generate in this study demonstrate the growing burden of PMAD and SMI among delivering women. They indicate that PMAD and SMI are associated with adverse obstetric outcomes, including SMMM, greater health care utilization, and more expensive deliveries to delivering women without these conditions. Racial disparities in SMI and PMAD diagnoses were evident and as well as those among women with Medicaid.

Study findings are consistent with other data indicating increases in PMAD over time. One previous study demonstrated a 65% increase in mental health conditions among perinatal women from in California [23]. Less research addresses SMI among obstetric populations, and it is evident not only that SMI is increasing among reproductive aged women, but also that women with SMI may be at increased risk of multiple adverse outcomes and costly deliveries. Previous work demonstrated rising incidence of SMMM [24] and preterm birth [25], and PMAD and SMI among delivering women may be contributing in part to these recent trends. Recent increases in the prevalence of mental health conditions among the general population [26, 27], and in particular among reproductive aged women [26, 28, 29], may partially explain the increased prevalence of PMAD observed given existing diagnoses may increase risk for PMAD and SMI-related episodes during the perinatal period [30].

In this nationally representative sample, white women had a higher prevalence of PMAD and SMI than non-Hispanic black and women of other races, and SMI were disproportionately higher among non-Hispanic black women compared to all other races. These results are consistent with a previous study from California [31] and among the general population [32], and may be driven by several unmeasured factors that contribute to racial/ethnic differences in the detection and prevalence of PMAD and SMI during the perinatal period. Differences in access, attitudes and stigma related to maternal mental health and SMI treatment, should be explored in future analyses.

Given that increases in PMAD and perinatal SMI are associated with adverse obstetric outcomes and higher delivery costs, these results underscore the burden perinatal mental health conditions place on both pregnant women and the healthcare system. For example, women with preexisting SMI are predisposed to relapse with psychotic breakdown at birth, necessitating psychiatric admissions [2, 30, 33], which may be one factor explaining their increased length of hospital stays and delivery costs. The Mental Health Parity and Addiction Equity Act of 2008 (MHPAEA) and the Affordable Care Act (ACA) of 2010 funded one of the largest expansions of mental health coverage [34], including an unprecedented opportunity to support comprehensive perinatal mental health treatment, which underscore the economic imperative to investigate whether these policies are reaching women most in need.

Although the study provides comprehensive national estimates of PMAD and SMI among pregnant women, there are several limitations inherent in using administrative datasets. The relative increases in prevalence of PMAD and SMI observed during the study period may be related to temporal increases in detection, yet the results likely underestimate the true burden, because they are based on codes from delivery records. Relatedly, the study data only included maternal mortality that occurred during the delivery hospitalization rather than after hospital discharge. Due to the cross-sectional design of the study data source that encompasses delivery hospitalizations, the prevalence of mental health conditions prior to or after delivery remains unknown, and the estimates do not reflect treatment or symptom remission. Lastly, due to the constraints of using a de-identified dataset, women may have given birth to more than one child over the study period, and thus, records may not reflect unique observations.

## Conclusion

This study documented a steady increase in the prevalence of PMAD and SMI among delivering women in the U.S. between 2006 and 2015. Publicly insured delivering women had a higher prevalence of SMI. Both PMAD and SMI were associated with SMMM, adverse obstetric outcomes, and more expensive deliveries. These national findings highlight the importance of addressing perinatal mental health conditions during pregnancy to prevent adverse obstetric outcomes including SMMM and contain delivery-related costs.

## Data Availability

The study included publicly available National Inpatient Sample (NIS) data from 2006 to 2015 administered by the Agency for Healthcare Research and Quality’s Healthcare Cost and Utilization Project available at: https://www.hcup-us.ahrq.gov/db/nation/nis/nisdbdocumentation.jsp [12].

## Abbreviations

PMAD: Perinatal mood and anxiety disorders
SMI: Serious mental illness
SMMM: Severe maternal morbidity and mortality
NIS: National Inpatient Sample
HCUP: Healthcare Cost and Utilization Project
USD: U.S. dollars
ACOG: American College of Obstetricians and Gynecologists
CI: Confidence interval
ICD-9-CM: International Classification of Diseases Ninth Revision, Clinical Modification Codes
